# SeroBA: rapid high-throughput serotyping of *Streptococcus pneumoniae* from whole genome sequence data

**DOI:** 10.1099/mgen.0.000186

**Published:** 2018-06-15

**Authors:** Lennard Epping, Andries J. van Tonder, Rebecca A. Gladstone, The Global Pneumococcal Sequencing Consortium,, Stephen D. Bentley, Andrew J. Page, Jacqueline A. Keane

**Affiliations:** ^1^​ Pathogen Informatics, Wellcome Sanger Institute, Hinxton, Cambridgeshire CB10 1SA, UK; ^2^​ Microbial Genomics, Robert Koch Institute, Berlin, Germany; ^3^​ Infection Genomics, Wellcome Sanger Institute, Hinxton, Cambridgeshire CB10 1SA, UK; ^4^​ Quadram Institute, Norwich Research Park, Norwich, UK

**Keywords:** *Streptococcus pneumoniae*, serotyping, pneumococcal, whole genome sequencing, *k*-mer method

## Abstract

*Streptococcus pneumoniae* is responsible for 240 000–460 000 deaths in children under 5 years of age each year. Accurate identification of pneumococcal serotypes is important for tracking the distribution and evolution of serotypes following the introduction of effective vaccines. Recent efforts have been made to infer serotypes directly from genomic data but current software approaches are limited and do not scale well. Here, we introduce a novel method, SeroBA, which uses a *k*-mer approach. We compare SeroBA against real and simulated data and present results on the concordance and computational performance against a validation dataset, the robustness and scalability when analysing a large dataset, and the impact of varying the depth of coverage on sequence-based serotyping. SeroBA can predict serotypes, by identifying the *cps* locus, directly from raw whole genome sequencing read data with 98 % concordance using a *k*-mer-based method, can process 10 000 samples in just over 1 day using a standard server and can call serotypes at a coverage as low as 15–21×. SeroBA is implemented in Python3 and is freely available under an open source GPLv3 licence from: https://github.com/sanger-pathogens/seroba

## Data Summary

1. The software is open source and available for Linux at Github under the GNU GPLv3 licence (url – https://github.com/sanger-pathogens/seroba).

2. Accession numbers for all sequencing reads and reference genomes that are used in the experiments are listed in the supplementary material (available in the online version of this article).

**Impact Statement**

This article describes SeroBA, a *k*-mer-based method for predicting the serotypes of *Streptococcus pneumoniae* from whole genome sequencing data. SeroBA can identify 92 serotypes and two subtypes with constant memory usage and low computational costs. We show that SeroBA is able to reliably predict serotypes at a coverage as low as between 15 and 21× and is scalable to large datasets.

## Introduction

*Streptococcus pneumoniae* (the pneumococcus) is a clinically important bacterium estimated to cause 700 000 to 1 million deaths in children under 5 years of age annually prior to the introduction of polysaccharide conjugate vaccines [1]. The capsular polysaccharide biosynthesis (*cps*) locus, which encodes the serotype, is a major virulence factor in *S. pneumoniae*. The introduction of multi-valent pneumococcal conjugate vaccines has led to a substantial change in the circulating serotypes [2] and decreased the number of deaths in children under 5 years of age to 240 000–460 000 annually [3]. However, serotype surveillance projects around the world showed an increase of *S. pneumoniae* disease due to non-vaccine serotypes that is caused by serotype replacement [4, 5]. Furthermore, it was observed that the serotype distribution differs between continents as well as single countries [6]. Therefore, it is very important to survey the circulating serotypes, in order to observe the epidemiological trends of *S. pneumoniae* before and after vaccination. The rapid reduction in the cost of whole genome sequencing (WGS) has led to its extensive use in the monitoring of pneumococcal serotypes [7].

To date, there are nearly 100 known serotypes described for *S. pneumoniae* based on differing biochemical and antigenic properties of the capsule [8]. The *cps* locus can be very similar between serotypes from the same serogroup (such as serogroup 6) with some of them distinguished by an SNP, rendering a gene non-functional or altering the sugar linkage [9]. However, dissimilar loci may be grouped in the same serogroup as they elicit a similar antibody response (e.g. serogroup 35). The large number of identified serotypes, and the high similarity between them, makes it challenging to computationally predict the serotype based on WGS data. Another challenge is recombination with other serotypes resulting in a mosaic *cps* locus [10], which may affect the polysaccharide being produced. It is possible to have significant variation across the *cps* locus which does not lead to a different polysaccharide capsule being produced [11]. Conversely, novel serotypes can be generated through these processes and can go unnoticed by antibody-based serotyping [12, 13]. Finally, mixed populations in a single sample and contamination can lead to ambiguity.

There are a number of methods available to predict serotypes in *S. pneumoniae*. Besides the gold standard method, Quellung, which can be subjective in certain cases, there are five additional methods based on serological tests, at least eight semi-automated molecular tests based on PCR and one method that uses microarray data for serotyping [14]. There are a number of *in-silico* methods to detect the *cps* locus, which can then be used to predict serotypes from WGS data [15–18]. However, the tool described by Metcalf *et al*. [18] is an in-house one, the tool described by Leung *et al*. [16] covers only half of the known serotypes, and the method from Croucher *et al.* [15] describes a mapping approach that is not implemented as an automated tool.

The only fully functional automated pipeline for serotyping *S. pneumoniae* WGS data is PneumoCaT, which was developed by Public Health England (PHE) [17]. PneumoCaT provides a capsular type variant (CTV) database including FASTA sequences for 92 serotypes and two subtypes as well as additional information about alleles, genes and SNPs for serotypes within specific serogroups. To predict a serotype, PneumoCaT uses bowtie2 [19] to align reads to all serotype sequences. If the serotype belongs to a predefined serogroup or the serotype sequence could not be unambiguously identified, PneumoCaT maps the reads to serogroup-specific genes to identify the genetic variants. However, it is computationally and memory intensive (Supplementary Material Section 3 Run time and Memory).

To address these problems, we developed SeroBA, which makes efficient use of computational resources in addition to accurately detecting the *cps* locus at low coverage, and we thus predict serotypes from WGS data using a database adapted from PneumoCaT [17]. Prediction accuracy was evaluated by comparing the results to a standard, validated dataset of 2065 samples from PHE [17]. We show that it is scalable and robust by calculating the serotypes of 9477 samples from the GPS (The Global Pneumococcal Sequencing) project, an ongoing global pneumococcal sequencing project, on commodity hardware. Simulated read data, generated from several reference genomes with varying coverage over the whole reference genome, were used to show the minimum depth of coverage required to call a serotype.

## Theory and implementation

**Fig. 1.**
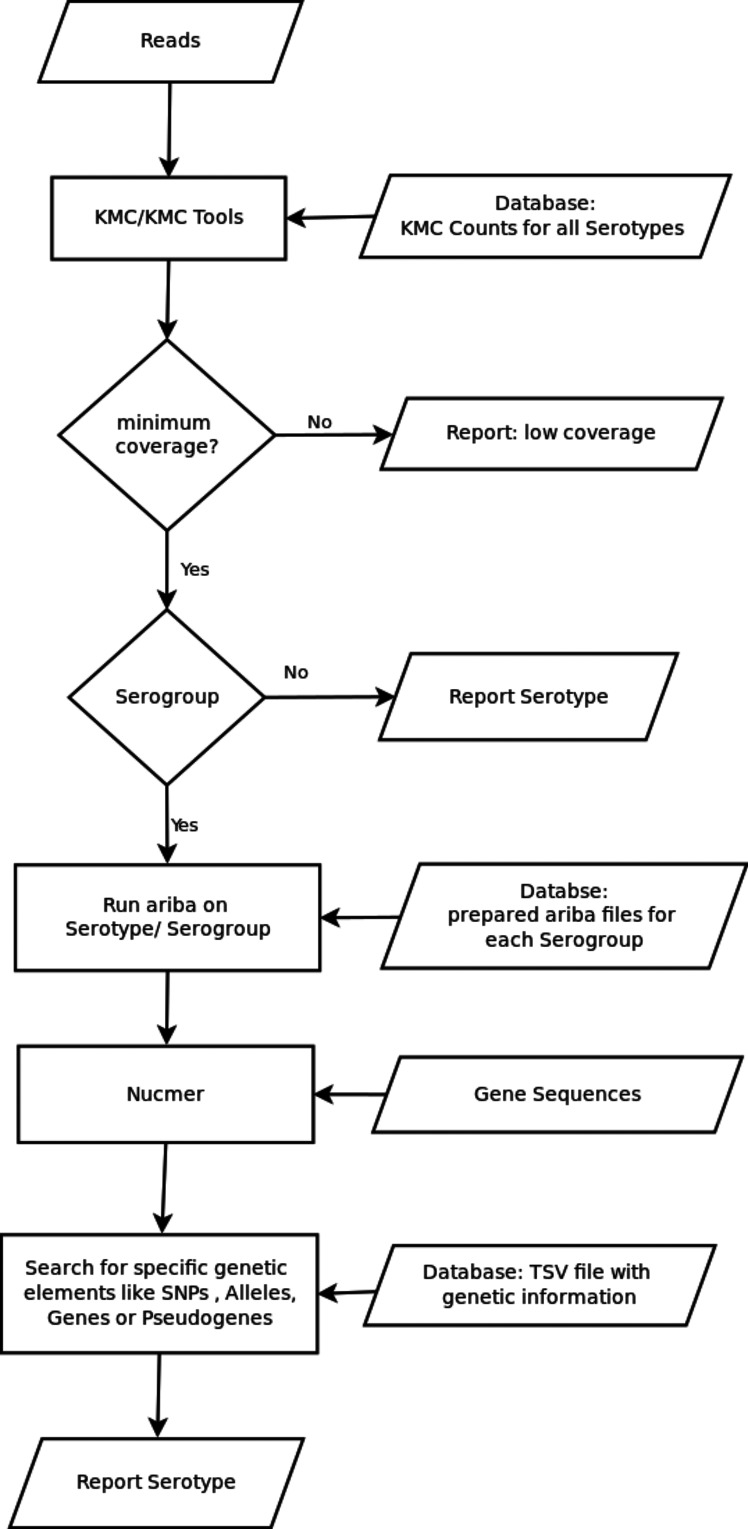
Flowchart outlining the main steps of the SeroBA algorithm.

SeroBA takes Illumina paired-end reads in FASTQ format as input as shown in Fig. 1. Precomputed databases that describe the serotypes are bundled with the SeroBA application. The first of these is a *k*-mer counts database for every serotype sequence. The *k*-mer counts database is generated using KMC (v3.0.0) [20] with a default *k*-mer size of 71 as this is the most resource-efficient size (Supplementary Material Section 2 Impact of K-mer Size, Figs S1 and S2). It is possible to vary the *k*-mer size using a user-defined parameter when generating the *k*-mer counts database. The second databse is an ARIBA- (v2.9.3) [21] compatible database for every serotype where serotypes are clustered together by their serogroup, and the third database is a CTV database, including FASTA sequences for 92 serotypes and two subtypes, as well as additional information about alleles, genes and SNPs for serotypes in specific serogroups. These databases were adapted from PneumoCaT [17]. A *k*-mer analysis is performed on all forward input reads, and the intersection is found between these *k*-mers and the precomputed *k*-mer database of serotypes by the use of the built-in intersection function of KMC. The *k*-mer coverage of the input reads over the serotype sequences is normalized by dividing the *k*-mer count on each serotype by its reference sequence length. The serotype with the highest normalized sequence coverage is selected. This step identifies the possible serotype or serogroup. At this stage 31 out of 92 serotypes can be identified without further computation (see Fig. S3). As this is done by a greedy algorithm, the serotype that was analysed first is taken in the event of a tie, although this is most likely to happen for serotypes within the same serogroup and will not lead to a misprediction. ARIBA is then used to build an assembly and to confirm the presence of the selected serotype from the raw reads. If a serogroup is selected, the *cps* sequence produced by ARIBA and serotype-specific genes are aligned with NUCmer [22] with parameters set as: min_id=90, min_length=200, maxmatch=True, show_snps=True, show_snps_C=False. This is done to find specific variants, such as presence/absence of genes, SNPs or gene truncations as defined in the CTV database. A gene is defined as present in the assembly if it has a minimum sequence similarity of 90 % and an alignment coverage of 95 %. The output of SeroBA includes the predicted serotype with detailed information that led to the prediction, as well as an assembly of the *cps* locus sequences.

## Validation dataset

A validation dataset consisting of 2065 UK isolates (Table S1) retrieved from the PHE archive was originally used to evaluate PneumoCaT. It consists of 72 out of 92 known serotypes, including all serotypes contained in commercial vaccines, and 19 non-typeable samples. The serotype of each sample was confirmed by latex agglutination with Statens Serum Institut typing sera [17]. PneumoCaT v1.1 [17] and SeroBA v0.1 with a *k*-mer size of 71 were evaluated on an AMD Opteron 6272 server running Ubuntu 12.04.2 LTS, with 32 cores and 256 GB of RAM. A single CPU (central processing unit) was used for each sample. A total of 25 of the 72 serotypes covered by the validation set can be directly predicted by the *k*-mer approach of SeroBA and of the 2065 isolates in the dataset 1881 were identified correctly by the *k*-mer approach.

**Fig. 2.**
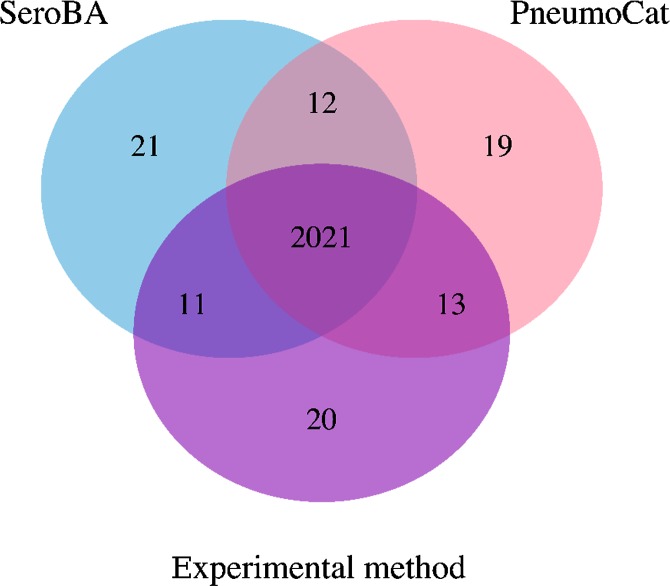
Agreement of serotyping results between different methods.

**Fig. 3.**
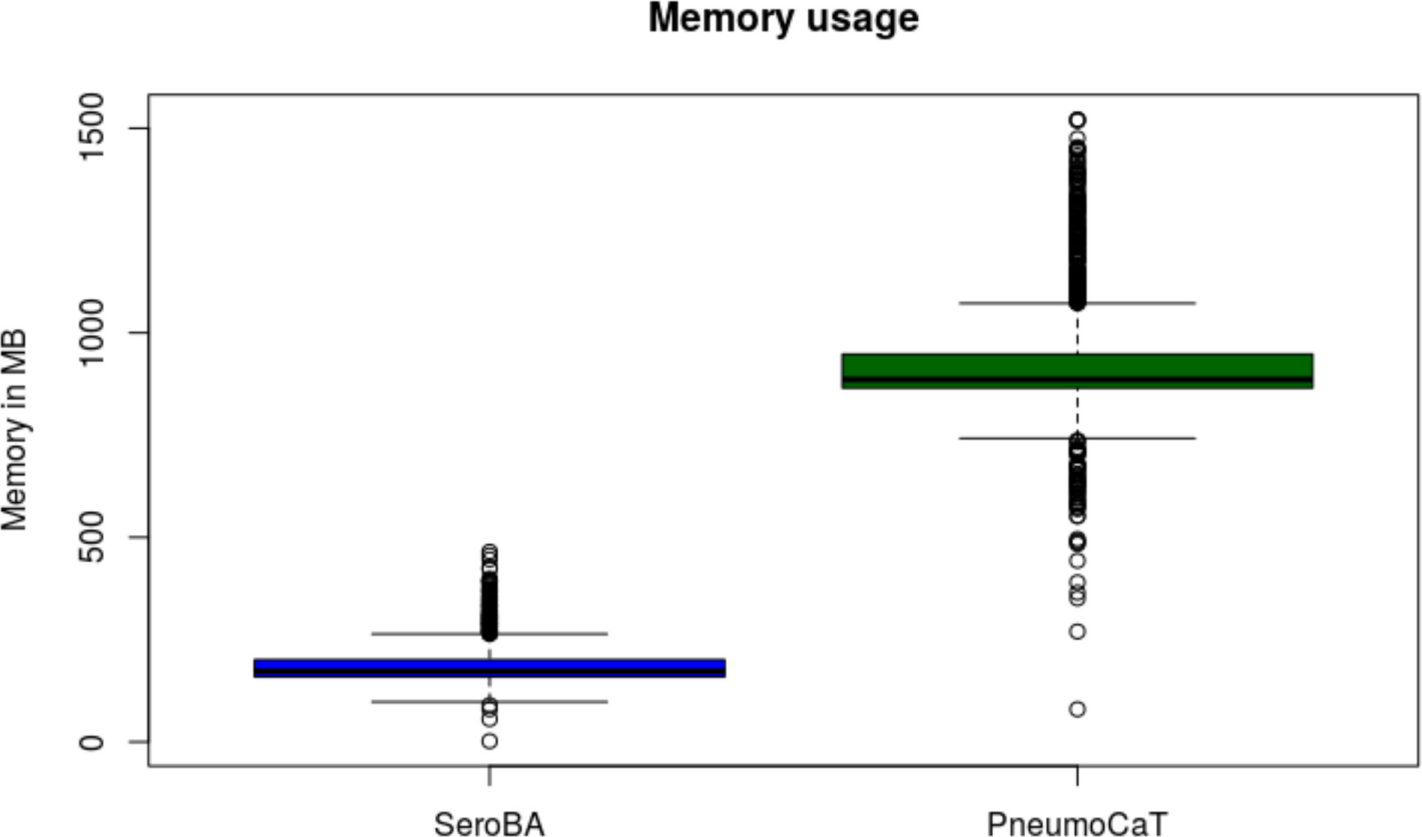
Memory usage of SeroBA and PneumoCaT on the validation dataset.

**Fig. 4.**
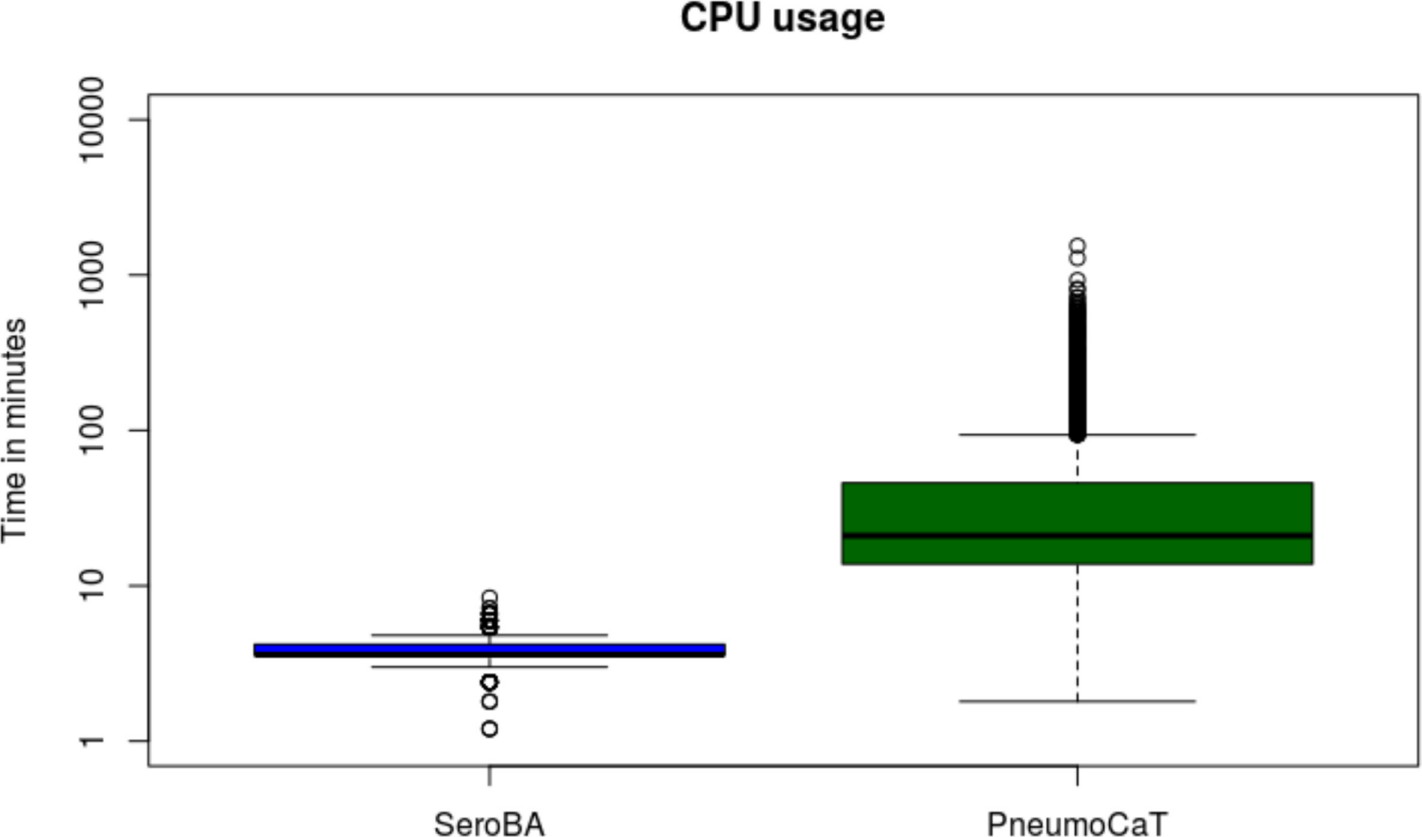
CPU usage of SeroBA and PneumoCaT on the validation dataset in minutes (log scale).

Fig. 2 summarizes the serotypes called for each sample by each method. As serotyping with latex agglutination and Quellung can be subjective [23] and potentially imprecise, a serotype was said to be concordant if two or more methods agreed on the same serotype. This gave a concordance of 98.4 % for SeroBA and 98.5 % for PneumoCaT with the latex agglutination method. Table S2 gives an overview of discordance between both computational methods and latex agglutination per serotype. The reference sequences in the CTV database for serotypes 24A, 24B and 24F may not be representative for the circulating strains [17], so SeroBA will report serogroup 24 instead of reporting the serotype. As discussed by Kapatai and others [17], serological prediction in serogoup 12 was error-prone, so a prediction of either serotype 12B or 12F was counted as concordant. The overall computational resources required to call the serotypes differed substantially between PneumoCaT and SeroBA (Figs 3 and 4 and Table S3): SeroBA was 15 times faster and required five times less memory than PneumoCaT.

We also calculated the sensitivity and specificity of SeroBA and PneumoCaT. For this, we took 41 publicly available samples, 33 *Streptococcus mitis* samples and eight *Streptococcus pseudopneumoniae* samples, as negative controls (Table S4). SeroBA did not predict any serotype for the negative control samples, whereas PneumoCaT predicted serotype 37 for three samples. In combination with the validation dataset we calculated a sensitivity and specificity of 0.98 and 1, respectively, for SeroBA and 0.98 and 0.92 for PneumoCaT (Tables S5 and S6). Further details on this can found in the supplementary material (Section 6 Sensitivity and Specificity).

## Evaluation using a large dataset

To show the scalability of SeroBA to large datasets, we took 9477 *S. pneumoniae* samples from the GPS project (Table S7) covering 74 serotypes and calculated the serotypes using the setup previously described, including a default *k*-mer size of 71. A comparison with serotypes determined using experimental methods gave an accuracy of 98.6 % for SeroBA. Details of the discordance between methods per serotype are given in Table S8. The serotypes were determined by different experimental methods as listed in Table S7. Using all 32 cores resulted in a total CPU time of 823.78 h. This showed that SeroBA can robustly scale to large datasets.

## Impact of depth of coverage

The effect of depth of coverage on the serotyping results produced by SeroBA and PneumoCaT was evaluated by simulating Illumina paired end reads from several reference genomes covering serotypes 1, 3, 4, 5, 6B, 19A, 19F and 23F (Table S9). Reads with a length of 250 bp were generated by DWGSIM (https://github.com/nh13/DWGSIM) using a fragment size of 500 bases, standard deviation of 50 and an error rate of 0.02. Coverage was increased from 1× to 50× in single steps and from 50× to 100× in steps of 10. Each experiment was repeated 10 times and the read depth at which SeroBA and PneumoCaT correctly predicted the serotype in 90 % or more of the experiments was noted as the minimum read depth required to correctly predict the serotype (Tables S10 and S11). In addition, the median values for memory and CPU time were calculated. SeroBA was used with a *k*-mer size of 51 and accurately predicted the serotype at a lower depth of coverage than PneumoCaT for six of the eight serotypes evaluated and started to predict the serotype at a depth of coverage of 18× for serotype 19A while PneumoCaT required 44× coverage. Fig. S4 shows that the computational resources required by SeroBA increases linearly at a lower rate than required by PneumoCaT. The amount of memory required by SeroBA stabilized at 150 MB, regardless of coverage, whereas PneumoCaT’s memory requirement increased as the depth of coverage increased, requiring four times more than SeroBA at 100× coverage.

## Conclusion

In this paper, we have described SeroBA, a method for predicting serotypes from *S. pneumonia*e Illumina Next Generation Sequencing (NGS) reads. We compared SeroBA and PneumoCaT with a gold standard experimental serotyping method (Quelling) and showed that they had approximately the same level of concordance. However, SeroBA was 15 times faster and required five times less memory than PneumoCaT. One of the main sources of error were samples with mosaic serotypes. SeroBA cannot automatically detect mosaic serotypes, but they can be manually identified by inspecting the assemblies provided by SeroBA and using a blast approach on the whole genome assembly to analyse the *cps* locus sequence. Furthermore, the assemblies of the *cps* locus sequence provided by SeroBA are very useful for other analyses. They can be used to detect novel mutations within a serogroup or to investigate the evolution of the *cps* locus for a set of *S. pneumoniae* samples by building a phylogenetic tree. SeroBA was able to predict the serotype from only 15–21× coverage and scaled well on a large dataset of nearly 10 000 samples with a prediction accuracy of over 98 %. Furthermore, we showed with negative control samples from *S. mitis* and *S. pseudopneumoniae* that SeroBA had a specificity of 100 % whereas PneumoCaT achieved 92 %.

## Data bibliography

1. Johnston C.H.G. *et al.* Genbank FJ440136.1 (2008).
2. Aslett M. *et al.* Genbank GCF_000211015.1 (2010).
3. Tettelin H. *et al*. Genbank GCF_000006885.1 (2001).
4. Hotopp J.D. *et al*. Genbank GCF_000018965.1 (2007).
5. Eli L. *et al.* Genbank GCA_001234125.1 (2001).
6. Donner J. *et al.* Genbank CP018136 (2016).
7. Mulas L. *et al.* Genbank GCF_000019825.1 (2008).
8. Croucher N.J. *et al.* Genbank FM211187 (2008).

## Supplementary Data

Supplementary File 1

## Supplementary Data

Supplementary File 2

## Supplementary Data

Supplementary File 3
