# Corrigendum: SeroBA: rapid high-throughput serotyping of *Streptococcus pneumoniae* from whole genome sequence data

**DOI:** 10.1099/mgen.0.000204

**Published:** 2018-08-31

**Authors:** Lennard Epping, Andries J. van Tonder, Rebecca A. Gladstone, The Global Pneumococcal Sequencing Consortium, Stephen D. Bentley, Andrew J. Page, Jacqueline A. Keane, David Aanensen, Maria-Cristina C. Brandileone, Martin Antonio, Samanta C.G. Almeida, Francisco Campos, P.E. Carter, Stuart C. Clarke, Jennifer Cornick, Nicholas Croucher, Ron Dagan, Sanjay H. Doiphode, Eric S. Donkor, Egorova Ekaterina, Ozgen Koseoglu Eser, Everett Dean, Rebecca Ford, Rebecca A. Gladstone, Anne Von Gottberg, Md. Hasanuzzaman, Paulina Hawkins, PL Ho, Waleria Hryniewicz, Andrew J. Pollard, Tamara Kastrin, Keith P. Klugman, Brenda Kwambana-Adams, Pierra Law, Deborah Lehmann, Thomas M. Lietman, Naima El Mdaghri, Benild Moiane, Helio Mucavele, Stephen K. Obaro, Theresa J. Ochoa, Metka Paragi, Tall Haoua, Mignon du Plessis, Rama Kandasamy, Nurit Porat, K. L. Ravikumar, Mabel Regueira, Ewa Sadowy, Samir K. Saha, Sadia Shakoor, Betuel Sigauque, Anna Skoczyoska, Kwan Soo Ko, Peggy-Estelle Tientcheu, Leonid P. Titov, Paul Turner, Balaji Veeraraghavan, Nicole Wolter, Stephen D. Bentley, Lesley McGee, Robert F. Breiman

**Affiliations:** ^1^​ Pathogen Informatics, Wellcome Sanger Institute, Hinxton, Cambridgeshire CB10 1SA, UK; ^2^​ Microbial Genomics, Robert Koch Institute, Berlin, Germany; ^3^​ Infection Genomics, Wellcome Sanger Institute, Hinxton, Cambridgeshire CB10 1SA, UK; ^4^​ Quadram Institute, Norwich Research Park, Norwich, UK

The members of The Global Pneumococcal Sequencing Consortium were not correctly acknowledged in the published article. The Acknowledgements should read as shown below.

## Acknowledgements

We thank Martin Hunt and the Infection Genomics and Pathogen Informatics groups at the Wellcome Trust Sanger Institute for testing and feedback during development. Furthermore, we wish to thank the authors of PneumoCaT for building the CTV database.

We also thank The Global Pneumococcal Sequencing Consortium. Members of The Global Pneumococcal Sequencing Consortium are David Aanensen, Maria-Cristina C. Brandileone, Martin Antonio, Samanta C. G. Almeida, Francisco Campos, P. E. Carter, Stuart C. Clarke, Jennifer Cornick, Nicholas Croucher, Ron Dagan, Dr Sanjay H. Doiphode, Sr. Consultant; Eric S. Donkor, PhD; Egorova Ekaterina, PhD; Ozgen Koseoglu Eser, Dean Everett, Rebecca Ford, Rebecca A. Gladstone, Anne Von Gottberg, Md. Hasanuzzaman, Paulina Hawkins, PL Ho, Waleria Hryniewicz, Andrew J. Pollard, FRCPCH PhD FMedSci Professor of Paediatric Infection and Immunity; Tamara Kastrin, Keith P. Klugman, Brenda Kwambana-Adams, Pierra Law, Deborah Lehmann, Thomas M Lietman, Naima El Mdaghri, Benild Moiane, Helio Mucavele, Stephen K. Obaro, MD Theresa J. Ochoa, Metka Paragi, Tall Haoua PharmD, MPH; Mignon du Plessis, Rama Kandasamy, Nurit Porat, Dr K. L. Ravikumar, Professor Emeritus; Mabel Regueira, Ewa Sadowy, Samir K. Saha, Sadia Shakoor, Assistant professor; Betuel Sigauque, Anna Skoczyoska, Kwan Soo Ko, PhD; Peggy-Estelle Tientcheu, Leonid P. Titov, Paul Turner, Balaji Veeraraghavan, Nicole Wolter, Stephen D. Bentley, Lesley McGee and Robert F. Breiman.

