# Supplementary material for: SeroBA: rapid high-throughput serotyping of Streptococcus pneumoniae from whole genome sequence data
Source: Microb Genom. 2018 Jun 15;4(7):e000186. doi: 10.1099/mgen.0.000186 (PMC6113868; doi:10.1099/mgen.0.000186)
Supplement: Supplementary File 1 [file mgen-4-186-s001.pdf]

Supplementary Material of:

SeroBA: rapid high-throughput serotyping of Streptococcus pneumoniae from whole genome sequence data

Authors: Lennard Epping[1,2,*], Andries J. van Tonder[3], Rebecca A. Gladstone[3], The Global Pneumococcal Sequencing consortium, Stephen D. Bentley[3], Andrew J. Page[1,4], Jacqueline A. Keane[1]

[1]Pathogen Informatics, Wellcome Trust Sanger Institute, Hinxton, Cambs, UK, CB10 1SA.
[2]Microbial Genomics, Robert Koch Institute, Berlin, Germany
[3]Infection Genomics, Wellcome Trust Sanger Institute, Hinxton, Cambs, UK, CB10 1SA.
[4] Quadram Institute, Norwich Research Park, Norwich, UK

[*]Corresponding email: eppingl@rki.de

1. Methods 3
1.1 KMC 3
1.2 Ariba 3
2. Impact of K-mer Size 4
3. SeroBA Decision Tree 5
4. Run time and Memory 7
5. Impact of Depth of Coverage 7
6. Sensitivity and Specificity 10
7. Causes of Discordance 11
7.1 Example of Discordant Result 11
8. Example Output 12
References 13

1. Methods

SeroBA makes use of two software applications and details of these are provided below.

1.1 KMC

KMC 3 (Kokot, Dlugosz, and Deorowicz 2017) is a fast and memory efficient tool to count *k*-mers. The *k*-mer count algorithm from KMC 3 uses two steps to process the input sequence data. First, it splits up the *k*-mers into bins, based on hash values, saving the intermediate results on disk. In the second stage, these bins are sorted with a parallelised version of radix sort for large datasets. The fact that the bins are stored on disk rather than kept in memory is the key reason why KMC 3 is so memory efficient. KMC tools provides a variety of operations to filter and manipulated the *k*-mer database e.g. different filter options, intersections and unions of two databases, complex set operations, or just providing statistics of a database. For SeroBA the intersection functionality from KMC tools is used to identify similar *k*-mers from two databases and the histogram option is used to analyse the *k*-mer depth of coverage.
To compare the two databases in SeroBA all *k*-mers from the reference files are used and from the input FASTQ files only *k*-mers that appear more than 3 times are used. This filters out unique *k*-mers which are often the result of sequencing errors and excludes low abundance *k*-mers which fall below the minimum depth of coverage required for *de novo* assembly. Therefore the KMC parameters are set as *"-ci4 -m1 -t1"*. The intersection between the read *k*-mer counts and each reference sequences *k*-mer counts is performed by *"kmc_tools simple 'kmer_count_reads' 'kmer_count_serotype' intersect temp_output"*. The number of intersections is normalized by dividing by the individual reference sequences length.

1.2 Ariba

ARIBA (Hunt et al. 2017) was originally developed to identify antimicrobial resistance associated genes and SNPs from short read Illumina data. First, ARIBA precomputes clusters using CD-hit (W. Li and Godzik 2006) on a set of reference sequences. The clusters are used as a database for the upcoming steps. The pre-clustering of the reference sequence makes it possible to analyse the database clusterwise rather than sequence by sequence. If metadata is provided, ARIBA performs a validation check, verifying if a coding sequence starts with a start codon and ends with a stop codon. Based on this clustering, ARIBA uses minimap (H. Li 2016) to approximately place reads on each reference sequence to build a set of reads for each cluster. The short read assembler fermi-lite (https://github.com/lh3/fermi-lite) provides a reference based assembly for each cluster, derived from the read set. This assembly is mapped using NUCmer (v3.0) (Kurtz et al. 2004) against all reference sequences in the corresponding cluster, to determine the closest reference. To identify SNPs, truncated genes and possible heterozygous SNPs, the reads are remapped to the assembly using bowtie2 (Langmead et al. 2009) . Variants are then called with the help of SAMtools (H. Li et al. 2009). The resulting information from these steps is stored in a tab delimited file and is provided, together with the assembly, as an output of ARIBA.
For SeroBA an ARIBA database that contains the clustered reference sequence and gene alleles for each serogroup is precomputed. To run ARIBA on a serogroup it is called with *"ariba run serotype_database read_1.fastq.gz read_2.fastq.gz output_directory"*

2. Impact of K-mer Size

We tested the effect of varying the *k*-mer size using the values 11, 23, 31, 51, 71 and 91. We selected 8 samples from the validation dataset covering 8 different serotypes that were already used to evaluate the impact of read depth. Each sample had sequence data with a read length of 250 base pairs and a read depth of over 100X. For all of the samples, the correct result was predicted from *k*-mer size ranging from 11 to 71. However, for k-mer size 91, SeroBA starts to become less concordant and erroneously called 3 of the samples as “untypable”. Using a *k*-mer size from 11 to 71 does not have any impact on the predicted result, however it does change the memory requirements of SeroBA from 350 MB to less than 200 MB on average (Supplementary Figures S1 and S2).

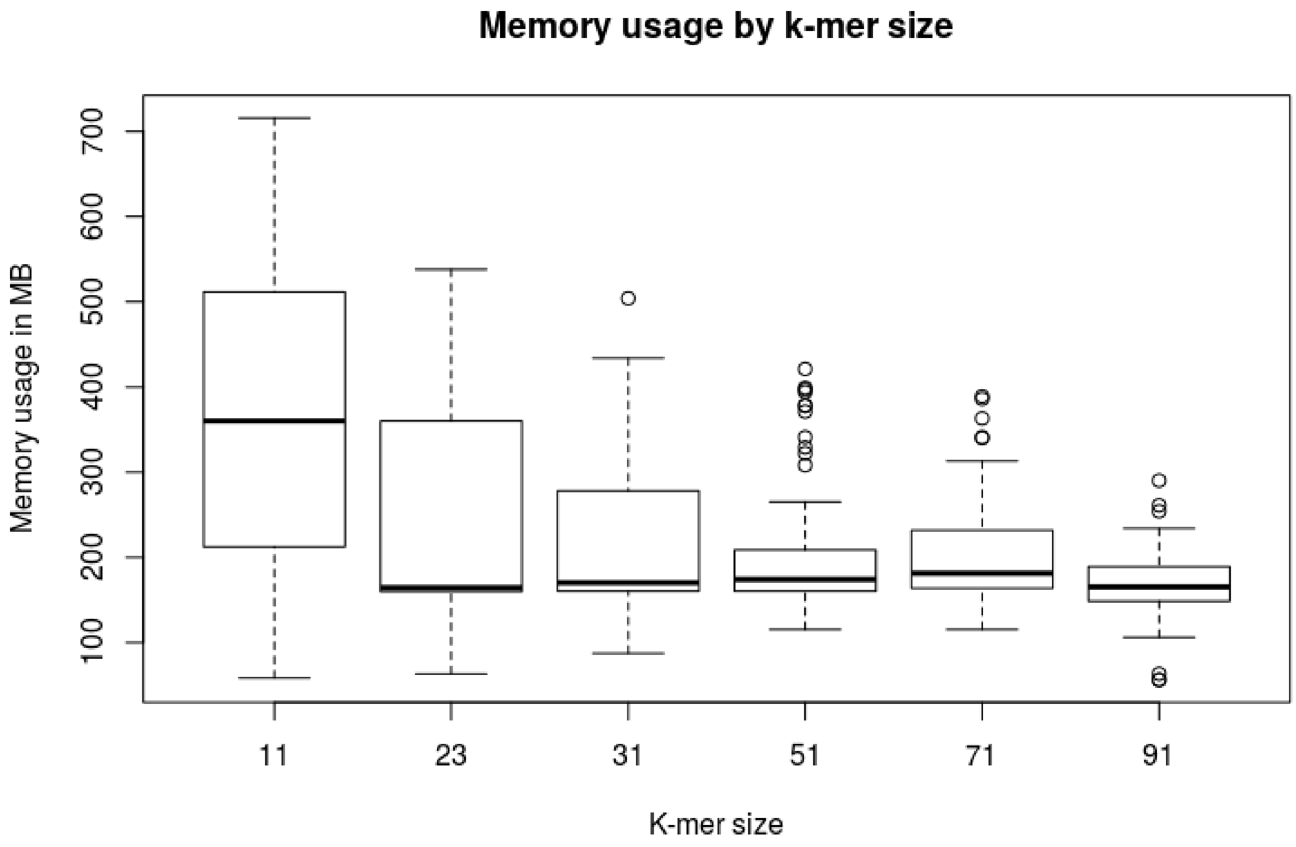

Supplementary Figure S1: Box plot of memory usage of SeroBA for different *k*-mer sizes.

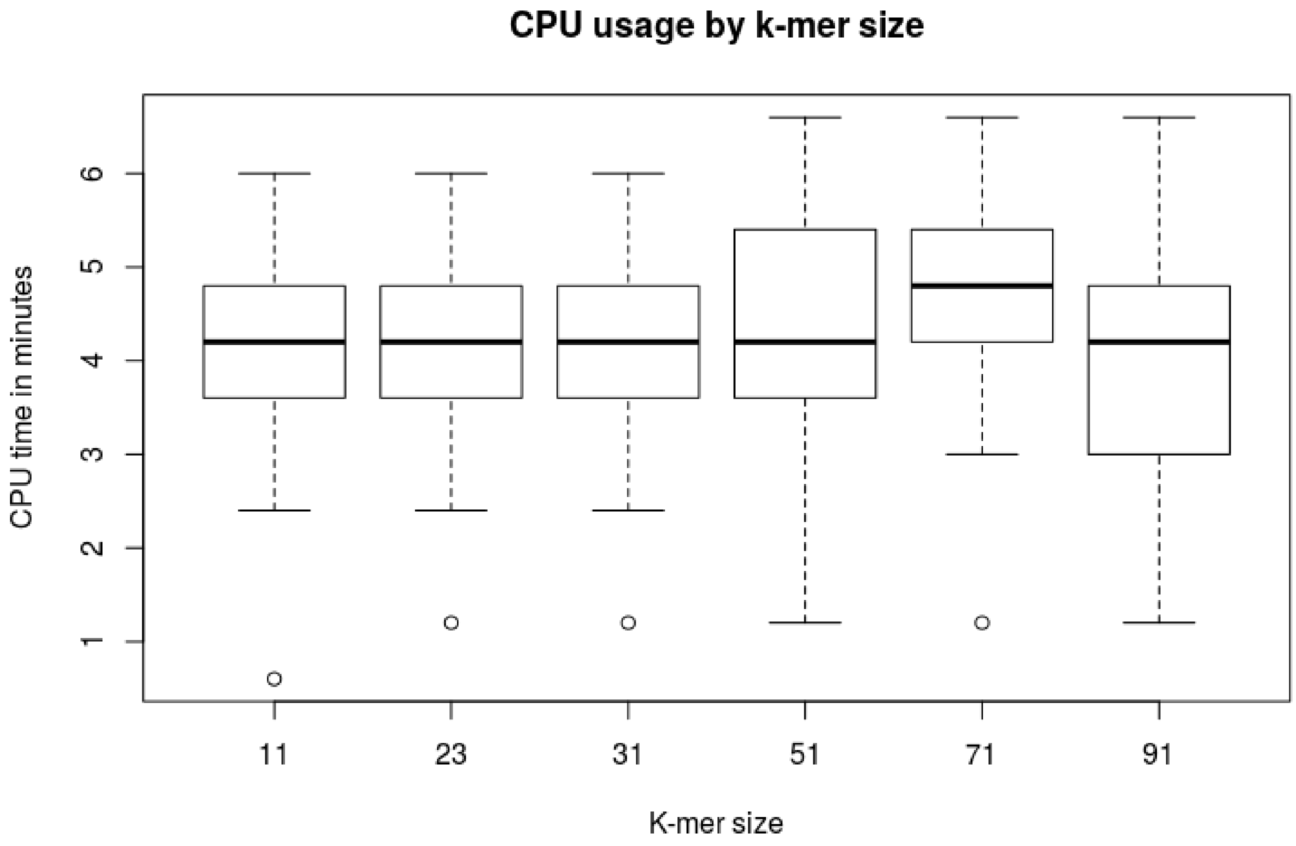

Supplementary Figure S2: Box plot of CPU usage of SeroBA for different *k*-mer sizes.

3. SeroBA Decision Tree

In general 34 of 92 serotypes can be distinguished by the *k*-mer approach used in SeroBA. For the remaining 58 serotypes further computation is required. The full decision tree is shown in Supplementary Figure S3.

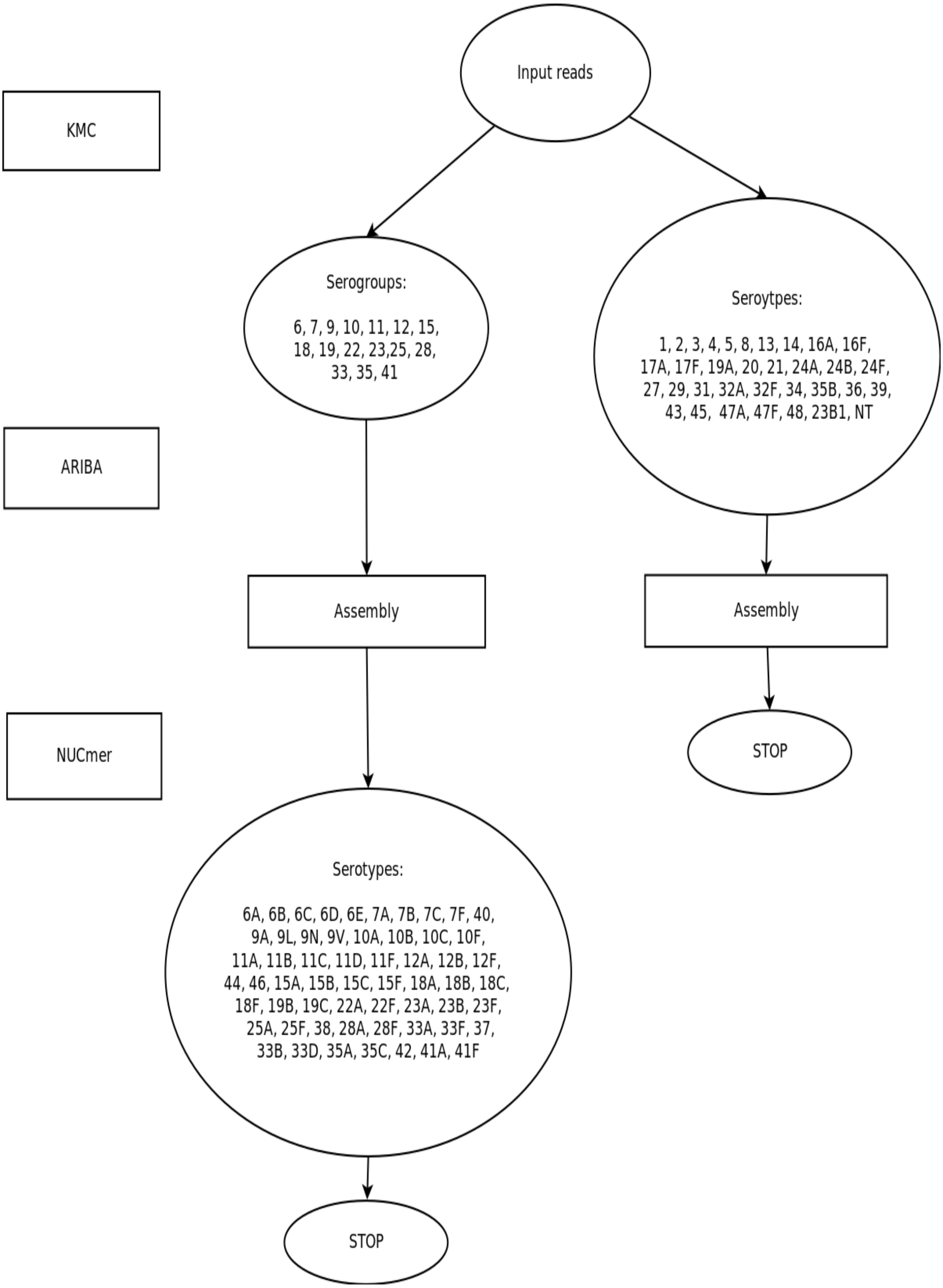

Supplementary Figure S3: This figure shows what serotype can be predicted at each stage of SeroBA. For further information of the different variants in each serogroup, please see (Kapatai et al. 2016) in Table 3.

4. Run time and Memory

Box plots of the memory and cpu usage of SeroBA and PneumoCaT are shown in Figures 3 and 4 for the validation dataset containing 2,065 samples. The mean memory and cpu usage for this validation set are also shown in Supplementary Table S3. This experiment was conducted on an AMD Opteron 6272 server running Ubuntu 12.04.2 LTS, with 32 cores and 256GB of RAM. A single CPU (Central Processing Unit) was used for each sample, repeated 10 times, with the mean memory usage and CPU times noted. These values were measured using the UNIX command time –v.

Additional Information for Figure 3 and 4:
In the box plots, the coloured areas represent the performance of 50% of the samples (50% quantile) with the thick black bar indicating the median of all data points. The upper and lower whiskers delimit the 25% and 75% quantile respectively. The black dots represent the outlier samples for both methods.

Supplementary Table S3: Performance of SeroBA and PneumoCaT on the validation set

Tool	Mean CPU time (m)	Mean RAM usage (MB)
PneumoCaT	65.84	922.89
SeroBA	4.53	187.82

5. Impact of Depth of Coverage

Supplementary Figure S2 shows the CPU and Memory usage for both methods, measured with the Unix command time –v, for each serotype with increasing read depth. The figure shows that the computational resources required by SeroBA grows linearly at a lower rate than PneumoCaT. This can be explained by the differences in the underlying algorithms used by the two approaches. In the first step the memory usage of SeroBA remains linear, because KMC stores the counted k-mers in bins on disk and keeps track of the data structure with a hashtable. In the second step, SeroBA makes use of ARIBA which down-samples the input reads to 50X which also keeps the memory requirements linear. PneumoCaT is using a standard mapping approach with bowtie2 that stores the input reads in memory. Furthermore, a k-mer approach just includes exact matches where read mapping with bowtie2 maps all reads with up to 2 mismatches which increases the possibilities of mapping position in the database exponentially and therefore leads to an increase in the total run time of a mapping approach with an increasing number of reads.

Supplementary Table S9 lists each serotype, its corresponding genome accession number and the minimum coverage that is required by SeroBA and PneumoCaT to predict the correct serotype.

Supplementary Table S9: This table shows the required coverage for SeroBA and PneumoCaT to predict the serotype based on simulated data generated from the reference genomes.

Serotype	Reference Accession	PneumoCaT Coverage	SeroBA Coverage
1	FJ440136.1	10	15
3	GCF_000211015.1	20	21
4	GCF_000006885.1	21	18
5	GCF_000018965.1	20	19
6B	GCA_001234125.1	25	20
19A	CP018136	44	18
19F	GCF_000019825.1	18	17
23F	FM211187	21	19

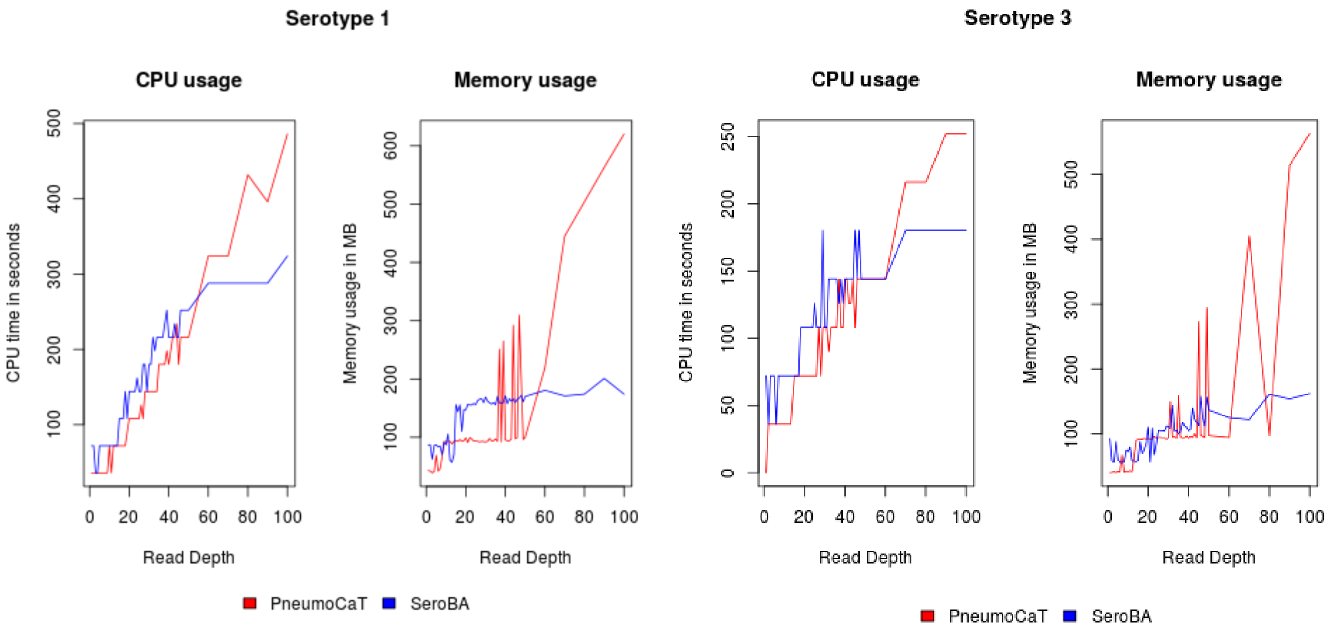

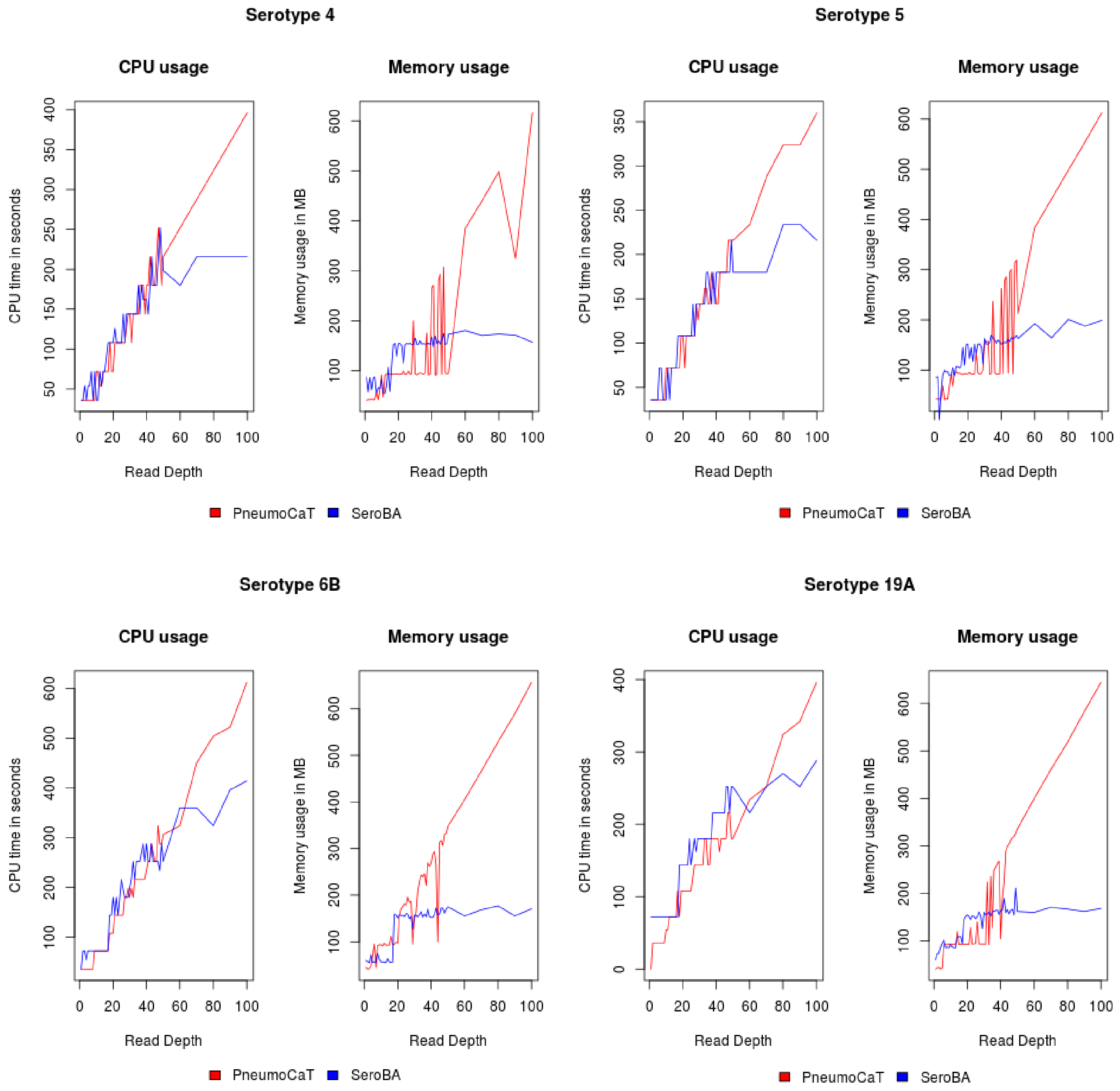
Serotype 4
CPU usage
Memory usage
Serotype 5
CPU usage
Memory usage
Serotype 6B
CPU usage
Memory usage
Serotype 19A
CPU usage
Memory usage
CPU time in seconds
Memory usage in MB
Read Depth
PneumoCaT
SeroBA

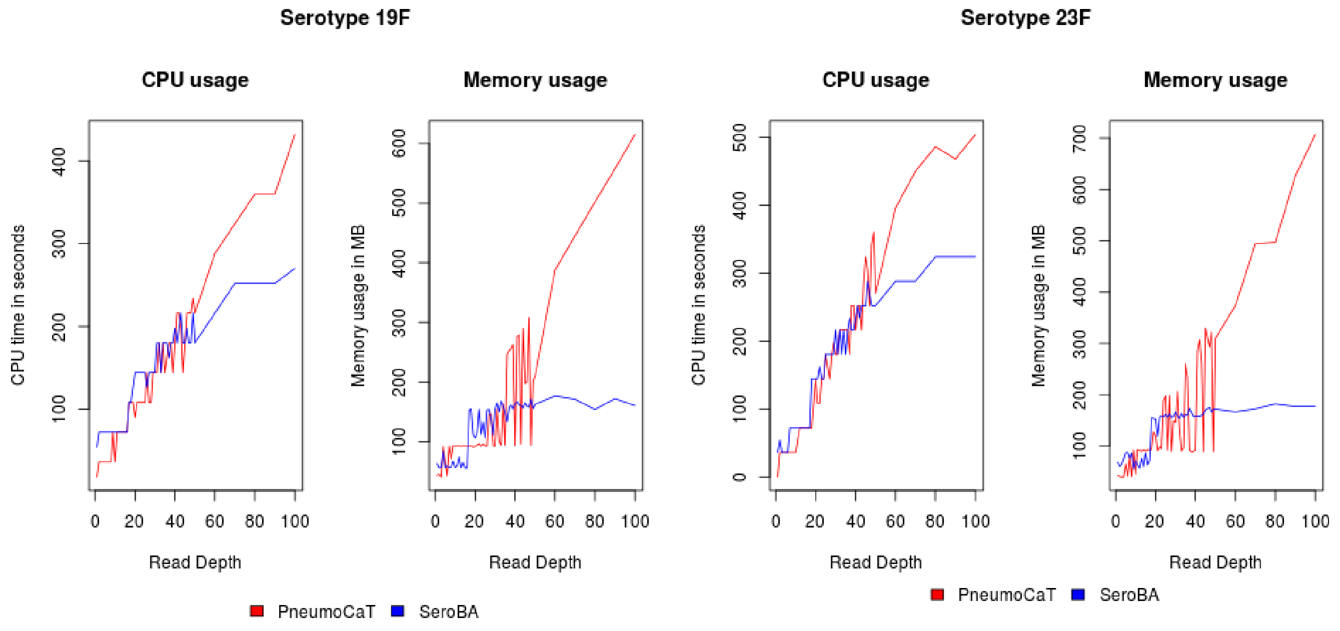

Supplementary Figure S4: These plots show the CPU and memory usage of SeroBA and PneumoCaT on 8 different serotypes with increasing read depth from 1X to 100X.

6. Sensitivity and Specificity

To calculate the sensitivity and specificity for SeroBA and PneumoCaT we selected 41 publically available samples from the ENA (European Nucleotide Archive), 33 *Streptococcus mitis* samples and 8 *Streptococcus pseudopneumoniae* samples as negative controls (see Supplementary Table S4). SeroBA was run with a default k-mer size of 71. For SeroBA an “untypable” or “low coverage” report was counted as correct and for PneumoCaT “Failed” or “invalid contig” was counted as a correct prediction for the negative controls. SeroBA could not predict any serotype for the negative control samples, whereas PneumoCaT predicted serotype 37 for 3 samples. As positive samples, the 2,065 samples from the validation dataset were used. The resulting sensitivity and specificity is shown in Supplementary Table S6.

Supplementary Table S5: Summary of the absolute values for SeroBA and PneumoCaT on the validation dataset and negative control samples to calculate the Sensitivity and Specificity.

	**PneumoCaT**	**SeroBA**
No. of samples untypable with experimental method and predicted as untypable with the software* (TN)	38	41
No. of samples untypable with experimental method and a serotype predicted with the software* (FN)	3	0
No. of samples predicted as a ST with experimental method and correct serotype predicted with software * (TP)	2034	2032
No. of samples predicted as a ST with experimental method and incorrect serotype predicted with software* (excluding untypable predictions with the software*) (FP)	31	33

*software is either PneumoCaT or SeroBA and fills in the appropriate columns per software

Supplementary Table S6: Sensitivity and Specificity for SeroBA and PneumoCaT calculated on the validation dataset and negative control samples

	SeroBA	PneumoCaT
Sensitivity (TP/P)	0.98	0.98
Specificity (TN/N)	1	0.92

7. Causes of Discordance

This section provides details on how to troubleshoot and inspect the cps assemblies in the case of unusual serotype predictions. Furthermore, we show an example of a discordant result between the latex agglutination and SeroBA prediction that was later identified as a mosaic serotype.

- Case 1:
  SeroBA predicts 'untypable'. An 'untypable' prediction can either be a real 'untypable' strain or can be the result of other issues. Possible issues include bad quality input data, incorrect species identification or coverage of the genome is too low. In this case, the user should perform a QC of the sequencing data, looking specifically at the QC results for these issues.

- Case 2:
  Low alignment identity in the 'detailed_serogroup_info' file or missing parts in the assembly that can be detected in the ‘report.tsv’ file. This can be a hint for a mosaic serotype. A possible solution is to perform a blast search on the whole genome assembly for genes dexB and aliA to inspect the sequence manually in between those genes.

7.1 Example of Discordant Result

An example of a sample producing a discordant result is ERR1439890 from the validation dataset. Sample ERR1439890 was predicted as serotype 10F using the experimental methods and predicted as serotype 35 using SeroBA. On further manual inspection of the *cps* locus assembly produced by SeroBA it was determined that there were genes missing from the *cps* locus. This sample was later identified as the mosaic serotype 10X (serotype 10F + 35).

8. Example Output

Taking sample ERR1440193/1195572 from the validation dataset as an example, SeroBA can be used to predict the serotype of this sample using the following command:

```
seroba runSerotyping /path/to/seroba/database ERS1195572_1.fastq.gz
ERS1195572_2.fastq.gz ERS1195572
```

This produces a folder `ERS1195572` containing a pred.tsv file containing the serotype predicted by SeroBA. The file `pred.tsv` looks like:

```
ERS1195572      23F
```

In addition, a file called `detailed_serogroup_info.txt` is created which includes information about SNP, genes, and alleles that are found in the input reads. The file `detailed_serogroup_info.txt` looks like:

```
Predicted Serotype:   23F
Serotype predicted by ariba     :23F
assembly from ariba as an identity of:     99.77  with this serotype
Serotype    Genetic Variant
23F  allele     wchA
```

closest reference in that specific serogroup according to ARIBA. Furthermore, you can see the sequence identity between the sequence assembly and the reference sequence In the detailed information, you can see the final predicted serotype as well as the serotypes that had the.

The results can be summarized using the command:

```
seroba summary .
```

This produces a tsv file called `summary.tsv` consisting of three columns (sample ID, serotype, comments). Serotypes that do not match any reference are marked as "untypable".

```
ERS1195572 23F
```

References

Kokot, Marek, Maciej Dlugosz, and Sebastian Deorowicz. 2017. “KMC 3: Counting and Manipulating K-Mer Statistics.” *Bioinformatics* 33 (17): 2759–61.

Kurtz, Stefan, Adam Phillippy, Arthur L. Delcher, Michael Smoot, Martin Shumway, Corina Antonescu, and Steven L. Salzberg. 2004. “Versatile and Open Software for Comparing Large Genomes.” *Genome Biology* 5 (2): R12.

Langmead, Ben, Cole Trapnell, Mihai Pop, and Steven L. Salzberg. 2009. “Ultrafast and Memory-Efficient Alignment of Short DNA Sequences to the Human Genome.” *Genome Biology* 10 (3): R25.

Li, Heng. 2016. “Minimap and Miniasm: Fast Mapping and de Novo Assembly for Noisy Long Sequences.” *Bioinformatics* 32 (14): 2103–10.

Li, Heng, Bob Handsaker, Alec Wysoker, Tim Fennell, Jue Ruan, Nils Homer, Gabor Marth, Goncalo Abecasis, Richard Durbin, and 1000 Genome Project Data Processing Subgroup. 2009. “The Sequence Alignment/Map Format and SAMtools.” *Bioinformatics* 25 (16): 2078–79.

Li, W., and A. Godzik. 2006. “Cd-Hit: A Fast Program for Clustering and Comparing Large Sets of Protein or Nucleotide Sequences.” *Bioinformatics* 22 (13): 1658–59.
