# Supplementary material for: SeroBA: rapid high-throughput serotyping of Streptococcus pneumoniae from whole genome sequence data
Source: Microb Genom. 2018 Jun 15;4(7):e000186. doi: 10.1099/mgen.0.000186 (PMC6113868; doi:10.1099/mgen.0.000186)
Supplement: Supplementary File 3 [file mgen-4-186-s003.pdf]

# The Global Pneumococcal Sequencing consortium

David Aanensen[1]; Maria-Cristina C. Brandileone[2]; Martin Antonio[3]; Samanta C. G. Almeida[2]; Francisco Campos[4]; P. E. Carter[5]; Stuart C. Clarke[6]; Jennifer Cornick[7]; Nicholas Croucher[8]; Ron Dagan[9]; Dr.Sanjay H Doiphode, Sr.Consultan[10]; Eric S. Donkor, PhD[11]; Egorova Ekaterina, PhD[12]; Ozgen Koseoglu Eser[13]; Dean Everett[7]; Rebecca Ford[14]; Rebecca A. Gladstone[15] ; Anne Von Gottberg[16]; Md. Hasanuzzaman[17]; Paulina Hawkins[18]; PL Ho[19]; Waleria Hryniewicz[20]; Andrew J Pollard, FRCPCH PhD FMedSci Professor of Paediatric Infection and Immunity[21]; Tamara Kastrin[22]; Keith P. Klugman[23,24]; Brenda Kwambana-Adams[3]; Pierra Law[19]; Deborah Lehmann[25]; Thomas M Lietman[26]; Naima EL MDAGHRI[27] ; Benild Moiane[28]; Helio Mucavele[28] ; Stephen K. Obaro MD[29,30,31]; MD Theresa J. Ochoa[4]; Metka Paragi[22]; Tall Haoua PharmD, MPH[32]; Mignon du Plessis[16]; Rama Kandasamy[21]; Nurit Porat[9]; Dr.K.L Ravikumar, Prof Emeritus[33];Mabel Regueira[34]; Ewa Sadowy[20] ; Samir K Saha[17]; Sadia Shakoor, Assistant professor[35]; Betuel Sigauque[28]; Anna Skoczyńska[20]; Kwan Soo Ko, Phd[36]; Peggy-Estelle Tientcheu[3]; Leonid P Titov[37]; Paul Turner[38,39]; Balaji Veeraraghavan[40]; Nicole Wolter[16]; Stephen D. Bentley[15]; Lesley McGee[18]; Robert F. Breiman[23,41]

[1]Centre for Genomic Pathogen Surveillance, Wellcome Trust Sanger Institute, Hinxton, CB10 1SA, UK
[2]Laboratory for Meningitis, Pneumonia and Pneumococcal Infection, Center of Bacteriology, Institute Adolfo Lutz, São Paulo, Brazil
[3]Medical Research Council Unit The Gambia, Banjul, The Gambia
[4]Grupo Peruano de Investigacion en Neumococo (GPIN), Universidad Peruana Cayetano Heredia, Lima, Peru
[5]Institute of Environmental Science and Research, Porirua, New Zealand
[6]Faculty of Medicine and Institute for Life Sciences and Global Health Research Institute, University of Southampton, Southampton SO17 1BJ, UK
[7]Malawi Liverpool Wellcome Trust Clinical Research Programme, Blantyre, Malawi
[8]Department of Infectious Disease Epidemiology, Imperial College London, London, UK
[9]Faculty of Health Sciences, Ben-Gurion University of the Negev, Beer-Sheva, Israel
[10]Microbiology Division, Department of Lab Medicine, Hamad Medical Corporation, Doha, Qatar
[11]Dept. of Medical Microbiology, University of Ghana, Accra, Ghana
[12]Laboratory of Clinical Microbiology and Biotechnology, Moscow Research Institute for Epidemiology and Microbiology, named after G. N. Gabrichevsky(MRIEM)
[13]Department of Medical Microbiology, Faculty of Medicine, Hacettepe University, Ankara, Turkey
[14]Papua New Guinea Institute of Medical Research, PO Box 60, Goroka, 441 Eastern Highlands Province, Papua New Guinea
[15]Wellcome Trust Sanger Institute, Hinxton, CB10 1SA, UK
[16]Centre for Respiratory Diseases and Meningitis, National Institute for Communicable Diseases, National Health Laboratory Service, Johannesburg, South Africa
[17]Child Health Research Foundation, Department of Microbiology, Dhaka Shishu Hospital, Dhaka, Bangladesh
[18]Centers for Disease Control and Prevention, Atlanta, USA

[19]Department of Microbiology, Queen Mary Hospital and Carol Yu Centre for Infection, The University of Hong Kong, Pokfulam, Hong Kong Special Administrative Region
[20]Department of Epidemiology and Clinical Microbiology, National Medicines Institute, Chełmska 30/34, 00-725 Warsaw, Poland
[21]Department of Paediatrics, University of Oxford, Room 02-46-07 Level 2, Children's Hospital, Oxford, OX3 9DU, United Kingdom
[22]Department for Public Health Microbiology, Centre for Medical Microbiology, National Laboratory of Health, Environment and Food, Grablovičeva 44, 1000, Ljubljana, Slovenia
[23]Department of Global Health, School of Public Health, Emory University, Atlanta, GA, USA
[24]Respiratory and Meningeal Pathogens Research Unit, Faculty of Medicine, University of the Witwatersrand, Johannesburg, South Africa
[25]Wesfarmers Centre of Vaccines and Infectious Diseases, Telethon Kids Institute, The University of Western Australia, Perth, Australia
[26]Francis I Proctor Foundation, University of California, San Francisco, California
[27]Laboratoire de Microbiologie, Faculté de médecine et de pharmacie, Université Hassan II, Casablanca, Maroc
[28]Centro de Investigação em Saúde da Manhiça, Maputo, Mozambique
[29]University of Nebraska Medical Center Omaha, NE
[30]Bayero University and Aminu Kano Teaching Hospital Kano
[31]International Foundation Against Infectious Disease in Nigeria (IFAIN)
[32]Agence de Médecine Préventive (AMP), 10 BP 638 Ouagadougou 10, Burkina Faso
[33]Dept. of Microbiology, Chief- Central Research Laboratory, KIMS Hospital and Research Centre, Bangalore
[34]Instituto Nacional de Enfermedades Infecciosas, ANLIS "Dr. Carlos G. Malbrán", Argentina
[35]Department of Pathology & Laboratory Medicine, Department of Pediatrics & Child Health, Aga Khan University, Karachi 74800, Pakistan
[36]Department of Molecular Cell Biology, Sungkyunkwan University School of Medicine, Suwon, South Korea
[37]Research-Practical Center for Epidemiology and Microbiology, Minsk. Belarus
[38]Cambodia-Oxford Medical Research Unit, Angkor Hospital for Children, Siem Reap, Cambodia
[39]Centre for Tropical Medicine and Global Health, Nuffield Department of Medicine, University of Oxford, Oxford, UK
[40]Christian Medical College, Vellore, India
[41]Emory Global Health Institute, Emory University, Atlanta, USA
